# Efficacy of laser interstitial thermal therapy for biopsy-proven radiation necrosis in radiographically recurrent brain metastases

**DOI:** 10.1093/noajnl/vdad031

**Published:** 2023-03-28

**Authors:** Michael Chan, Steven Tatter, Veronica Chiang, Peter Fecci, Roy Strowd, Sujit Prabhu, Constantinos Hadjipanayis, John Kirkpatrick, David Sun, Kaylyn Sinicrope, Alireza M Mohammadi, Parag Sevak, Steven Abram, Albert H Kim, Eric Leuthardt, Samuel Chao, John Phillips, Michel Lacroix, Brian Williams, Dimitris Placantonakis, Joshua Silverman, James Baumgartner, David Piccioni, Adrian Laxton

**Affiliations:** Wake Forest Baptist Health, Winston-Salem, North Carolina, USA; Wake Forest Baptist Health, Winston-Salem, North Carolina, USA; Yale School of Medicine, New Haven, Connecticut, USA; Duke University Medical Center, Durham, North Carolina, USA; Wake Forest Baptist Health, Winston-Salem, North Carolina, USA; The University of Texas MD Anderson Cancer Center, Houston, Texas, USA; University of Pittsburgh School of Medicine, Pittsburgh, Pennsylvania, USA; Duke University Medical Center, Durham, North Carolina, USA; Norton Neuroscience Institute, Louisville, Kentucky, USA; Norton Neuroscience Institute, Louisville, Kentucky, USA; Cleveland Clinic Lerner College of Medicine at CWRU, Cleveland, Ohio, USA; Norton Neuroscience Institute, Louisville, Kentucky, USA; Ascension St. Thomas Hospital West, Nashville, Tennessee, USA; Washington University School of Medicine, St. Louis, Missouri, USA; Washington University School of Medicine, St. Louis, Missouri, USA; Cleveland Clinic Lerner College of Medicine at CWRU, Cleveland, Ohio, USA; Ascension St. Thomas Hospital West, Nashville, Tennessee, USA; Geisinger Medical Center, Danville, Pennsylvania, USA; University of Louisville Health, Louisville, Kentucky, USA; NYU Grossman School of Medicine, New York, New York, USA; NYU Grossman School of Medicine, New York, New York, USA; AdventHealth Medical Group, Orlando, Florida, USA; University of California San Diego Health, La Jolla, California, USA; Wake Forest Baptist Health, Winston-Salem, North Carolina, USA

**Keywords:** brain metastasis, Laser interstitial thermal therapy (LITT), radiographic progression, radiation necrosis (RN), stereotactic laser ablation (SLA)

## Abstract

**Background:**

Laser interstitial thermal therapy (LITT) in the setting of post-SRS radiation necrosis (RN) for patients with brain metastases has growing evidence for efficacy. However, questions remain regarding hospitalization, local control, symptom control, and concurrent use of therapies.

**Methods:**

Demographics, intraprocedural data, safety, Karnofsky performance status (KPS), and survival data were prospectively collected and then analyzed on patients who consented between 2016–2020 and who were undergoing LITT for biopsy-proven RN at one of 14 US centers. Data were monitored for accuracy. Statistical analysis included individual variable summaries, multivariable Fine and Gray analysis, and Kaplan–Meier estimated survival.

**Results:**

Ninety patients met the inclusion criteria. Four patients underwent 2 ablations on the same day. Median hospitalization time was 32.5 hours. The median time to corticosteroid cessation after LITT was 13.0 days (0.0, 1229.0) and cumulative incidence of lesional progression was 19% at 1 year. Median post-procedure overall survival was 2.55 years [1.66, infinity] and 77.1% at one year as estimated by KaplanMeier. Median KPS remained at 80 through 2-year follow-up. Seizure prevalence was 12% within 1-month post-LITT and 7.9% at 3 months; down from 34.4% within 60-day prior to procedure.

**Conclusions:**

LITT for RN was not only again found to be safe with low patient morbidity but was also a highly effective treatment for RN for both local control and symptom management (including seizures). In addition to averting expected neurological death, LITT facilitates ongoing systemic therapy (in particular immunotherapy) by enabling the rapid cessation of steroids, thereby facilitating maximal possible survival for these patients.

**Key Points**

- Due to fast postoperative recovery, patients typically have short hospital stays.
- Laser interstitial thermal therapy (LITT) can improve radiation necrosis (RN) symptoms, allowing quick corticosteroid tapering.
- Consistent with previous publications, LITT provides durable control of RN.

**Importance of the Study**

This large, prospective, and multicenter cohort confirms and expands upon the utilization for laser interstitial thermal therapy (LITT) in the treatment of stereotactic radiosurgery-induced radiation necrosis (RN). Even in the setting of subtotal ablation, LITT provides a durable response in pathologically proven RN with 75.7% of patients never experiencing disease progression of the ablated lesion prior to death nor requiring further treatment during the study follow-up. No patients died of RN in this series and seizure control was improved after LITT. In addition to LITT facilitating rapid taper-off corticosteroids, these data also illustrate that patients can continue receiving systemic therapy such as cytotoxic chemotherapy and immunotherapy with little to no interruption after the LITT procedure. Lastly, given the mounting evidence for the efficacy of LITT in treating RN, this may facilitate considering more aggressive use of radiotherapy to avoid tumor recurrence after SRS treatment of brain metastases.

The use of stereotactic radiosurgery (SRS) for the treatment of brain metastases has increased dramatically in the past decade due to the publication of studies showing superior cognitive outcomes as compared to whole-brain radiation,^1,2^ an emerging literature for treating larger^3^ and more numerous^4^ metastases, and greater community access to this technology.^5^ With the expanded use of SRS, there has been a corresponding increased prevalence of post-SRS imaging changes, or radiographic progression, which can represent either radiation necrosis (RN) or tumor progression. Either of these conditions can occur anytime from months to years after SRS.^6^

A clinical dilemma arising from the increasing cases of post-SRS imaging changes stems from the fact that conventional MRI cannot reliably distinguish between RN and tumor progression.^7^ While pathologic confirmation remains a gold standard option for patients experiencing imaging changes post-SRS, there are some clear disadvantages of craniotomy such as wound healing time leading to systemic treatment delay, hospitalization time, infection risk, and risk of operating in close proximity to eloquent brain. Alternatively, a less aggressive approach of serial imaging and allowing the biology to declare itself may lead to poorer outcomes in patients who ultimately progress to larger or symptomatic metastases.^8^ A watchful-waiting approach that also utilizes steroids can place a patient at risk for steroid-induced effects such as myopathy and immune suppression that can potentially limit future treatment options, particularly for those patients on immunotherapy.^9^

Laser interstitial thermal therapy (LITT) is an increasingly utilized tool in the setting of post-SRS imaging changes due to its minimally invasive nature, its same-procedure pathologic confirmation of RN versus tumor progression, low complication rate, and its ability to therapeutically intervene for either diagnosis.^10,11^ A recent retrospective series of 75 patients demonstrated that the local control outcomes of LITT were comparable to those of craniotomy for both RN and tumor progression.^12^ LITT compared with medical management for RN also showed that LITT resulted in a significant reduction in lesion size at the 10–12 months time point whereas medical management had no such effect.^13^ Recent retrospective analyses suggest LITT should be considered for first-line treatment of progressive disease with compelling outcomes in both recurrent tumors and RN.^13,14^ Early evidence also suggests there may be negative clinical and survival implications associated with delaying intervention in this patient population.^8,13,14^ There are also emerging signals in both preclinical and clinical models that LITT helps facilitate immune cell recruitment and activation within the CNS and that LITT may augment the effects of checkpoint blockade inhibitors,^15–17^ potentially benefiting the overall treatment of brain metastases and improving local control even when directed at RN. Given these encouraging results and remaining questions, efficacy of LITT in the setting of post-SRS radiographic progression for patients with brain metastases was explored through a comprehensive analysis of the prospective, multicenter Laser Ablation of Abnormal Neurological Tissue Using Robotic NeuroBlate System (LAANTERN) study (NCT02392078).

## Materials and Methods

### Patient Enrollment

LAANTERN is an IRB-approved, multisite, prospective study currently enrolled across 26 institutions in the United States. LAANTERN allows for follow-up data to be captured for up to 5 years following the LITT procedure. Details pertaining to the LAANTERN study were previously described.^11,18–20^ Monitoring with source verification and data management were enacted to ensure the accuracy of the deidentified data in the electronic database. The study protocol was reviewed and approved by the institutional review board (IRB) at each participating center. Informed consent was obtained for all subjects using IRB-approved documentation.

Patients were included in this analysis if they had one or more radiographically progressive brain metastasis with biopsy-proven RN at time of LITT procedure, without evidence of tumor recurrence on pathology. Those with mixed pathology showing tumor and RN were excluded. Patients were also required to be eligible for 2-year follow-up, thus all patients underwent the LITT procedure prior to August 31, 2020 to ensure opportunity for prolonged data collection. All patients in the cohort have consented to enrollment between 2016 and 2020.

### Surgical Management

All centers used the FDA-cleared NeuroBlate® System (Monteris Medical, Minneapolis, MN) as previously described.^21^ Surgical preplanning, technique, and biopsy during the procedure were performed as standard of care at each institution.

### Variables Collected

The LAANTERN study collects demographic and health history information as well as disease-specific outcome measures. The following variables were collected and included in this analysis: demographics, diagnosis date, treatment prior to and after LITT, primary cancer type, tumor location, tumor size per physician measurement (captured as a 2 or 3-dimensional measurement), surgical “skin to skin” time, total laser ablation time and total energy applied to the lesion during ablation, extent of ablation per neurosurgeon estimation, Adverse events (AE), hospitalization data, post-procedure overall survival (OS) and local freedom from disease progression data on the target ablated lesion, neurologic assessment over time, Karnofsky Performance Scale (KPS) and Functional Assessment of Cancer Therapy—Brain (FACT-Br) over time. Baseline demographics and medical history was collected through 60-day prior to the LITT procedure. KPS and FACT-Br scoring was collected at baseline and at each follow-up visit.

For analysis of time to corticosteroid therapy cessation following LITT, patients were included if they had a corticosteroid start date within 6 weeks leading up to the ablation procedure or within 2 weeks after the procedure, with day-of-procedure counting as day 0 of steroid use.

Survival data were estimated using the Kaplan–Meier method.^22^ Local freedom from disease progression was defined as the time from the date of the LITT procedure on the target lesion to progression of the target lesion as defined by investigator-determined radiographic progression on MRI with recommended use of Response assessment in neuro-oncology brain metastases (RANO-BM) criteria. Post-procedure all-cause overall survival (OS) was defined as the time from LITT procedure to death.

AE were reviewed by an independent safety committee composed of neurosurgeons with laser ablation expertise. The committee reviewed site-reported AEs and adjudicated related events into neurological versus non-neurological categories. Neurological deficits were specified as temporary or chronic (defined as persisting for >30 days). Relatedness to the NeuroBlate system, LITT procedure, and surgical procedure itself were also adjudicated and are reported as described previously.^23^ Events were rated as mild, moderate, or severe. Severe events were defined as having had complex management requiring prolonged hospitalization or resulting in death. Moderate events were clinically significant or had uncomplicated management requiring hospitalization. Mild events were clinically inconsequential or required only outpatient management.

### Statistical Analysis

Categorical variables were summarized using relative frequencies and percentages. Continuous variables were summarized using mean ± standard deviation for data with normal distribution or median with interquartile range (IQR); 25th, 75th percentile or minimum and maximum. All variables were summarized at the individual level, except for lesion-specific variables including lesion depth, volume, percent ablated, and anatomical location, which were summarized at the lesion level. Lesion size was measured by the sites in 2 or 3 dimensions along the greatest length, width, and/or height. If 3-dimensional measurements were obtained, the ratio for the volume of a cube to the volume of a sphere was used and volume was calculated by V = (length × width × height)/2. If 2-dimensions were provided, the equation for the volume of a sphere was applied with the radius obtained from length and width measurements. If multiple lesions were ablated within the same subject, the earliest date of target-lesion progression was used for local freedom from disease progression analysis.

Median Survival and Kaplan–Meier Product Limit analyses were used to depict survival of patients. For KPS, the *P*-value from the Student’s *t*-test was used in a per-patient analysis to compare scores from each independent follow-up timepoint to baseline scores. A chi-squared test was used to compare the rates of hemorrhage between various subgroups of patients in this analysis and against reported rates in the literature. A multivariable Fine and Gray competing risk model was used to assess multivariate differences in disease progression (with death as a competing risk) after the procedure based on these variables: Age > 65, gender, lesion volume > 3.5 cc, 100% ablation, concurrent use of steroids, immunotherapy use, prior use of Whole-Brain Radiation (WBRT), and baseline KPS > 70. A Cox proportional hazard model was used to examine the risk of disease progression for near-total ablations and large lesions. Hazard ratios and Confidence intervals (CI) are reported where applicable. All reported *P*-values were 2-sided, and a *P*-value < 0.05 was considered statistically significant. All statistical analyses were performed using Stata version 17.

## Results

### Overall Demographics and Procedure

A total of 180 subjects with metastatic brain lesions targeted by LITT were identified. Of these, fourteen US centers enrolled 90 recurrent brain metastasis patients with biopsy-proven, pure RN who met the criteria for inclusion (Supplementary Figure 1). The median length of follow-up in this cohort was 1.65 years (range 0.02–4.18 years). Demographic details of the cohort are shown in Table 1. The median age of patients was 65 years (min 27, max 83) and 57.8% were female. Median baseline KPS was 90 and was > 70 in 75.3% of patients. Prior therapy included radiation (100%), resection (24.4%), immunotherapy (22.2%), and chemotherapy (36.7%). Targeted therapies for systemic cancer were not collected as part of this study; however, for those reporting molecular markers 8.9% had p53 mutations, 3.3% were estrogen receptor-positive, 2.2% expressed Her2, 2.2% had Kirsten rat sarcoma viral oncogene homolog (KRAS) mutation, and 2.2% had Breast Cancer gene 2 (BRCA2) mutations. Primary cancer is broken down in Table 1 with the majority being non-small cell lung cancer (NSCLC) (42.2%), breast cancer (10.0%), or melanoma (8.9%). There were 38.3% of lesions classified as “deep” and 45.7% classified as “superficial” with further breakdown of anatomical location seen in Table 1. Median lesion volume per physician-reported measurement was 3.64cc (Min/Max 0.04cc, 21.25cc, largest upper quartile of lesions was >7.35cc).

**Table 1.** Demographics

Characteristics and Measures	All Subjects (*N* = 90)
Age, mean (SD), y	63.3 (11.2)
Female, No. (%)	52 (57.8)
Race/ethnicity, No. (%) White	74 (82.2)
Black/African American	12 (13.3)
Asian	1 (1.1)
American Indian or Eskimo/Aleutian	1 (1.1)
Multiracial/Unknown	2 (2.2)
*Primary Cancer type, No. (%)*	
Non-small cell lung cancer (NSCLC)	38 (42.2)
Breast	9 (10.0)
Adenocarcinoma carcinoma unspecified or lung unspecified	8 (8.9)
Melanoma	8 (8.9)
Renal or Kidney	8 (8.9)
Ovarian	4 (4.4)
Small cell lung carcinoma (SCLC)	4 (4.4)
Other	11 (12.2)
Seizures at baseline, No. (%)	31 (34.4)
Number of Lesions, No.	94
*Anatomical Lesion location, No. (%)*	
Frontal Lobe	43 (45.7)
Temporal Lobe	17 (18.1)
Parietal Lobe	16 (17.0)
Occipital Lobe	10 (10.6)
Cerebellum	11 (11.7)
Corpus Callosum	1 (1.1)
Other	3 (3.2)
*Prior treatment and neoadjuvant therapy, No. (%)*	
LITT Ablation (any)	0 (0)
Resection	22 (24.4)
Partial resection (10%–50%)	1 (4.5)
Subtotal resection (51%–90%)	2 (9.1)
Near gross total resection (91%–99%)	4 (18.2)
Gross total resection (100%)	8 (36.4)
Immunotherapy	20 (22.2)
Chemotherapy	33 (36.7)
Radiation	90 (100.0)
Stereotactic	84 (93.3)
Whole-Brain Radiation (WBRT)	9 (10.0)
Local	7 (7.8)
Stereotactic and WBRT	9 (10.0)
Stereotactic and Local	1 (1.1)
WBRT and Local	0 (0.0)

#### Procedural safety and hospitalization

Table 2 summarizes procedural experiences and AE. The median total skin-to-skin procedure time was 165.5 minutes. Total laser time and total energy applied to the lesion were available on 60/90 patients. Median laser time was 10 minutess 16 sec (min 57 seconds, max 51 minutes) and median laser energy applied was 8769 KJ. Per physician report, 35.9% of lesions were completely ablated with full coverage of the lesion with thermal damage threshold lines, 47.8% were near-total ablations (91%–99% coverage), and 16.3% were subtotal ablated (51%–90% coverage). There were 4 patients who had 2 lesions ablated on the same-procedure day. Post-operatively, 38.6% of patients were transferred to the ICU and 61.3% of patients went to a step-down or standard floor. Patients remained in the hospital for a median time of 32.5 hours (range 9.4–436.9) and 87.8% were discharged to home from the hospital.

**Table 2.** Procedural Outcomes and Adverse Events

Characteristics and Measures	All subjects (*N* = 90)
Procedure time, mean (SD), hours	2.9 ± 1.1
Length of hospital stay, median (IQR), hours	32.5 (29.0, 54.3)
Transferred to ICU post-LITT, n/*N* (%)	34/88 (38.6)
Discharged to home, No. (%)	79 (87.8)
Total Adverse Events, No. (%)	17 (18.9)
Neurological Deficit	5 (5.6)
Motor	3 (3.3)
Speech aphasia	1 (1.1)
Blurry vision/Visual disturbance/Visual field deficit	1 (1.1)
Seizure—new type/new onset	1 (1.1)
Edema, symptomatic worsening	2 (2.2)
Hemorrhage, clinically significant	3 (3.3)
Hemorrhage, clinically insignificant	1 (1.1)
Cardiac related	1 (1.1)
Deep vein thrombosis	1 (1.1)
Hypoxia	1 (1.1)
Pneumonia	1 (1.1)
Removal of foreign object	1 (1.1)
LITT/surgery-related severe adverse events, no. (%)	2 (2.2)

Sites reported a total of 34 AEs, and all were adjudicated by the safety committee. No AEs were adjudicated as being caused by device or system malfunctions and 17 events were adjudicated as procedure-related (Table 2). The remaining 17 events were determined to not be related to the device or surgical procedure; 2 (2) events were severe, and both were related to disease progression (one reporting weakness and one reporting nausea/malaise). Neurological complications occurred in 5 patients (5.6%). All 5 events were considered chronic, which was defined as a deficit persisting for greater than 30 days. Three (3) patients experienced a chronic moderate motor deficit which included (1) worsened focal weakness, (1) postural lean, and (1) increased tone. One (1) patient with baseline lower left quadrant anopsia experienced homonymous hemianopsia following ablation of the right occipital lobe. One (1) patient with baseline aphasia (and dysarthria) experienced worsened aphasia described as garbled speech. Clinically significant hemorrhage was reported in 3.3% of patients. Of these hemorrhages, 2 (2) were reported as moderate requiring medical management only and one (1) was reported as severe requiring placement of a ventricular drain for intracranial pressure management and was due to the biopsy (occurring pre-LITT). The 2 moderate hemorrhages were identified post-LITT in patients who also had biopsies during the procedure. There were no deaths related to the procedure. One (1) patient required removal of a foreign object after screw tips from their navigation system became embedded in the patient’s skull.

#### Symptom management outcome

A baseline history of seizures was reported in 34.4% of patients. Seizure occurrence within 1-month post-LITT was reported in 12.0% of patients and seizure occurrence at 3 months was reported in 7.9% of patients. Only one patient had a new onset seizure which was reported as an adverse event. For those patients who indicated history of seizures at baseline and were on anti-seizure medications, the most common anti-seizure medication was Levetiracetam (Keppra) reported in 88% either alone or in combination with another drug (Gabapentin or brivaracetam combined use reported in 13.6%). Gabapentin alone was used in 8% and brivaracetam alone in 4%. After the LITT procedure, anti-seizure medication dosage was decreased or discontinued in 64% of patients (range 1 day to 2 years after the procedure), 28% reported no change in medications (same type, no dosage difference), and medication was increased in 8% (these patients (*N* = 2) were on Gabapentin alone or in combination with Levetiracetam).

**Figure 1.**
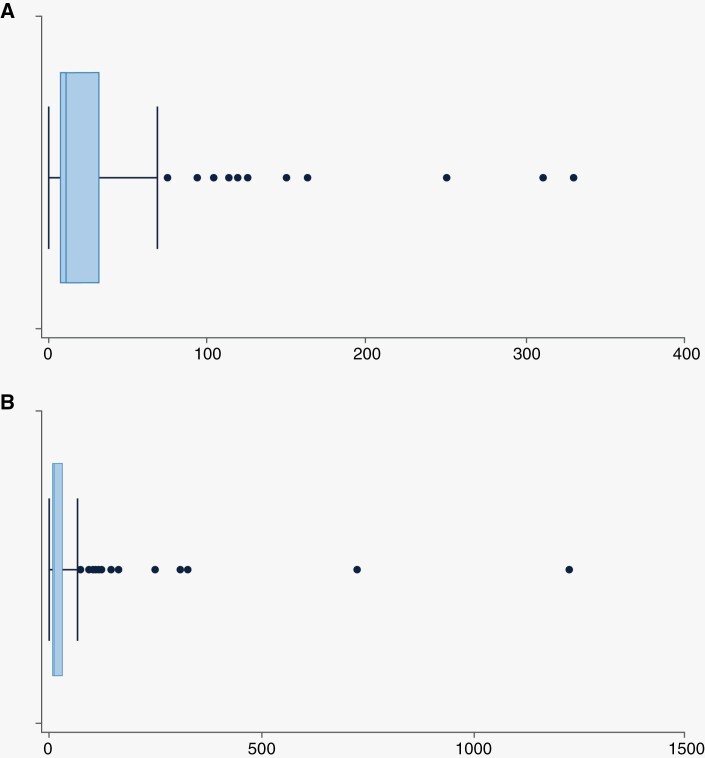
Box Plots of post-procedural time on steroids in days for patients started on steroids within 6 weeks prior to 2 weeks after the laser interstitial thermal therapy procedure. (A) presents steroid duration and (B) includes all outliers.

The median time to steroid cessation after undergoing LITT for RN was 13 days (min 0, max 1229, IQR 7, 33.0). These data are summarized in Table 3 and Figure 1A with all data including statistical outliers represented in Figure 1B. The most extreme outlier was on corticosteroids for 1229.0 days per the last follow-up report. This patient with NSCLC, diagnosed in 2012, had a recorded history of chronic daily corticosteroid use for 4 years prior to the LITT procedure for unreported reasons. At 30-day post-LITT, 26.6% of patients still taking corticosteroids reported a dose decrease even though they had not fully stopped their medication.

**Table 3.** Post-Procedural Adjunctive Therapy

Characteristics and Measures^*^	All Subjects (*N* = 90)
Time on Steroids after Procedure, days	*N* = 79
Mean (SD)	62.1 (166.8)
Median, (Min, Max)	13 (0.0, 1229.0)
Time from LITT to start of Chemotherapy, days	*N* = 32
Mean (SD)	154.9 ± 153.3
Median, (Min, Max)	91 (1.0, 488)
Never stopped chemotherapy, No. (%)	11/27 (40.7)
Time from LITT to start of Immunotherapy, days	*N* = 9
Mean (SD)	388.1 (383.4)
Median, (Min, Max)	302 (43, 1330)
Never stopped immunotherapy	3/17 (17.6)

^*^Baseline steroid use includes patients who were started on steroids within 6 weeks before or 2 weeks after Laser interstitial thermal therapy (LITT) procedure. Chemotherapy and immunotherapy baseline use was defined as therapy delivered within three months prior to the LITT procedure.

#### Re-initiation of systemic therapies

During the 6 weeks following the LITT procedure, no patients required a resection and 1 patient was reported to be treated with bevacizumab. Three (3/17) patients (17.6%) had no interruptions of their immunotherapy regimens despite surgery and 11/27 patients (40.7%) never stopped their chemotherapy regimens despite undergoing LITT (Table 3). One patient with the biopsy-related bleed still had LITT and was on continued chemotherapy without interruption. Reason for discontinuation of chemotherapy or immunotherapy was not reported in this study. No toxicity was noted in these patients from the combination of continuing therapy plus LITT. When comparing the rates of clinically significant hemorrhage in the group that never stopped therapy (1/14; 7.14%) versus those not on therapy at the time of LITT (2/76; 2.63%), there was no significant difference (*P* = .388). Nine subjects (9/32; 28%) had follow-up chemotherapy initiated within 6 weeks of LITT and the median time to follow-up chemotherapy for those patients was 23 days. No toxicity was noted in these patients. For those patients who did not continue therapy during the procedural interval, no patients had follow-up immunotherapy initiated within 6 weeks after the LITT procedure and eight patients (8/17) reported immunotherapy use at baseline that was not continued or reinitiated after LITT.

#### Progression-free and overall survival

**Figure 2.**
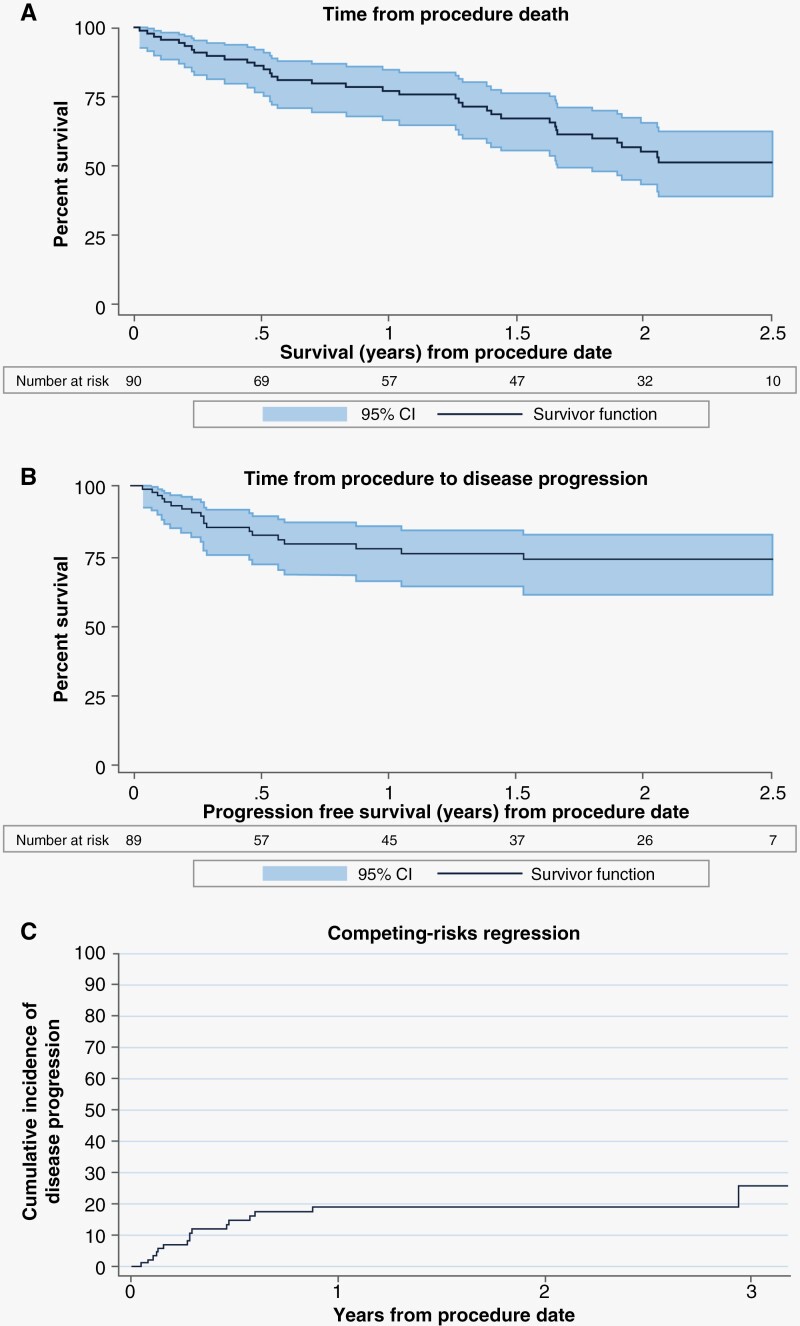
Estimated survival by Kaplan–Meier and Fine and Gray analysis. (A) estimated overall survival from time of procedure to death (all cause). (B) estimated freedom from local lesional progression (lesion treated with laser interstitial thermal therapy) from time of procedure. (C) Fine and Gray competing risk model for disease progression with death as a competing risk.

Median local freedom from disease progression was not yet reached in this cohort of 90 patients with a minimum of 24-month follow-up. Median post-procedure overall survival (OS) was 2.55 years (95% confidence interval [CI]: 1.66, not reached). In this cohort of 90 patients, 37 were reported to have died during the duration of follow-up time (min 24–max 60 months). Of these 37 deaths, 9 patients were shown to have disease progression of the ablated lesion prior to death and 28/37 (75.7%) never reported disease progression of the ablated lesion prior to death. Estimated OS by Kaplan Meier showed 77.1% of patients were alive at 1 year and 55.4% were alive at 2 years (Figure 2A). Freedom from local lesional progression was estimated as 77.5% at 1 year (Figure 2B). A multivariable Fine and Gray competing risk model for disease progression and death was performed and there were no significant variables identified in the analysis (Table 4). The cumulative incidence function of competing-risks regression is presented in Figure 2C. A multivariate analysis for all-cause death alone showed only baseline KPS > 70 correlated with freedom from death (HR 0.42 (CI 0.19, 0.94), *P* = .034). An additional analysis was performed to examine the risk of disease progression in those with more complete ablative coverage and those with larger lesions where there is potential for residual tissue. In those with total and near-total ablations (91% or greater ablative coverage) versus those with subtotal ablations (<90%) there was no significant difference in risk of disease progression (HR 0.83 (CI 0.24, 2.86), *P* = .763). Risk of disease progression based on lesion size (largest upper quartile of lesions >7.35cc versus all smaller lesions) was also performed and again did not detect significance (HR 1.46 (CI 0.55, 3.84), *P* = .443). When risk of progression in larger lesions with subtotal ablations were simultaneously examined, there was still no significant difference in risk of progression (*P* = .662 and *P* = .902, respectively).

**Table 4.** Multivariable Fine and Gray Competing Risk Model for Disease Progression (With Death as a Competing Risk)

Variable	Number of Patients	Sub-hazard Ratio (95% CI)	*P*-value
Age > 65 years	43	0.53 (0.16, 1.70)	.284
Female gender	52	1.77 (0.61, 5.13)	.290
Lesion volume > 3.5 cc	45	1.17 (0.38, 3.59)	.779
Extent of Ablation 100%	33	0.98 (0.33, 2.90)	.964
Concurrent use of steroids	72	1.14 (0.33, 3.91)	.839
Prior or concurrent immunotherapy use	20	0.80 (0.22, 2.90)	.732
Prior use of WBRT	9	0.44 (0.04, 4.60)	.496
Baseline KPS > 70	61	1.18 (0.29, 4.85)	.815

#### Functional assessment

Median KPS at baseline was 90 (50, 100). The median KPS remained at 80 (60, 100) through the 2-year follow-up mark. KPS over time with mean/median changes over time is displayed in Supplementary Table 1. FACT-Br Total Score median was 141.0 (95.0, 196.0) at baseline and the median remained above 140.0 throughout the entirety of the 2-year collection data (Supplementary Table 2). Baseline headaches were reported in 31.4% (27/86) of patients. Changes in headache status through 12 months are seen in Supplementary Table 3—in those with headaches at 1 month, 65% reported either no change or an improvement.

## Discussion

RN represents an inflammatory molecular cascade mediated by vascular injury and propagated by vascular endothelial growth factor (VEG-F) and cytokine release.^24^ The mechanism by which surgical resection mitigates this phenomenon is by physical removal of the nidus of the chemical cascade.^25^ In turn, LITT can ablate and denature this molecular nidus through the process of thermal ablation and in turn prevent its further propagation.^26^ Radiographically, RN can be indistinguishable from tumor progression. Multi-parametric MRI analysis such as dual-phase Positron Emission Tomography - Computed Tomography (PET-CT) and AI/Radiomics can help elucidate a diagnosis, but this can delay treatment decision-making and lacks the sensitivity of biopsy, which is considered the gold standard. LITT offers the ability to obtain concomitant intra-operative biopsy and to make real-time treatment decisions based on pathology. Likewise, LITT is able to successfully treat either biopsy result. Our results suggest that a complete ablation covering the entire lesion periphery is not necessarily required to achieve disruption of the nidus of the chemical cascade in patients with biopsy-confirmed RN. However, if tumor is present on biopsy, complete ablation should be the goal as previous studies have noted lesions with residual tumors are less likely to achieve complete response radiographically.^10^

Several prior reports exist demonstrating efficacy of LITT in the treatment of RN.^10,11,27^ The present series represents the largest prospectively gathered dataset for the use of LITT for the treatment of RN. In general, LITT is a highly effective treatment for pathologically proven RN. More than 75% of patients in the present series did not require any further treatment for the duration of follow-up in this study. These results are comparable to the reported efficacy of craniotomy.^12^ Patients in this analysis also experienced a reduction in seizure prevalence after the LITT procedure.

A major advantage of LITT over craniotomy is its decreased burden to patients by virtue of its minimal invasiveness. In the present series, the median hospitalization time was 32.5 hours. A recent large analysis of craniotomy outcomes for brain metastases cited a median stay nearly twice as long as the present series after craniotomy (3 days).^28^ Another advantage is that cancer patients receiving LITT can commonly continue receiving systemic therapy such as cytotoxic chemotherapy, targeted agents, and immunotherapy with LITT with little to no interruption, whereas craniotomy will commonly require several weeks for wound healing to occur before re-starting systemic agents. Patients on chemotherapy can have reduced platelet counts making them more prone to bleeding. Although there was one significant bleeding event that occurred due to needle biopsy in a patient on chemotherapy at the time of LITT, the risk was not statistically significant over the comparator group not continuing therapy (*P* = .388) or over the known 3% risk of unexpected or significant bleeding in patients undergoing brain surgery (*P* = .361).^29^

Several non-surgical strategies have been reported for the treatment of SRS-induced RN including corticosteroids, bevacizumab, hyperbaric oxygen, and pentoxifylline. While the use of corticosteroids has classically been considered the first-line treatment for RN, toxicities such as myopathy, diabetes, immunosuppression, and psychosis can significantly alter a patient’s health status and their ability to continue systemic therapies for their cancer and lack of response to corticosteroids is not infrequent. Bevacizumab is increasingly used to treat the symptoms of RN. Unlike corticosteroids, bevacizumab does alter the underlying pathobiology given its action on VEG-F, yet lesions frequently recur once the medication is stopped. Toxicities such as intestinal perforation, intracranial hemorrhage, and thromboembolic events are also possible and have led to a fatal adverse event rate of approximately 2.9%.^30^ A randomized trial of bevacizumab versus steroids for post-SRS RN was opened by the Alliance for Clinical Trials in Oncology but closed early due to lack of accrual. Two other retrospective studies have looked at LITT versus bevacizumab for brain metastasis patients with RN. One study (*N* = 25 LITT, *N* = 13 bevacizumab), a single-institution retrospective review, reported longer OS and better long-term lesional volume reduction for those who underwent LITT vs those treated with bevacizumab.^31^ The other, a systematic review and meta-analysis of 148 patients who underwent LITT and 143 patients who received bevacizumab, reported superior survival rates in the LITT cohort seen at 18 months with low rates of AE in both groups.^32^ When lesions were assessed by Radiological response (RANO) criteria, this same study showed LITT-targeted lesions were more likely to be stable (49.2%; *P* = .002) versus patients targeted with Bevacizumab reported a majority of lesions having only partial response (79.6%; *P* = .001) at six months or last follow-up.^32^ Patient selection bias and inconsistent use of biopsy to confirm a RN diagnosis are acknowledged in these studies. The survival results of this prospective study are consistent, if not potentially superior, with outcomes previously published for bevacizumab;^31,32^ however prospective controlled studies are needed to make stronger conclusions.

A population for which there may be an increasing utilization of LITT are those who are being treated with immunotherapy. For brain metastasis patients, immunotherapy has led to impressive advances in OS for multiple cancer histologies. In spite of a decreased likelihood of neurologic death,^33^ there are now several reports documenting the combination of SRS and immunotherapy leading to a greater likelihood of RN.^34–36^ Steroids are known to impair the efficacy of immunotherapies, which are increasingly being used for the treatment of metastatic cancer.^37^ A recent meta-analysis showed that daily steroid usage in brain metastasis patients treated with immunotherapy worsens OS and progression-free survival.^38^ Median time to steroid cessation in this analysis was 13 days and other recent publications cite LITT’s advantage in time to steroid independence for patients with RN.^10,13^ This ability to wean off steroids after LITT is reflective of excellent symptom management.

RN is not without patient risk and can accompany serious symptomatology that can decrease quality of life and even shorten survival time for patients. However, patients diagnosed with recurrent tumors generally have worse OS and progression-free survival outcomes than those with RN.^10,39^ Having effective, minimally invasive options for the management of RN could allow for the more aggressive use of radiotherapy, offering expanded treatment options to patients with newly diagnosed and recurrent metastatic disease. Laser ablation is recommended in the NCCN (National Comprehensive Cancer Network) guidelines Version 2.2022 as a consideration for patients with recurrent metastatic disease, RN, and recurrent glioblastoma.^40^ The neurosurgical medical specialty societies (AANS/CNS) have recently published an evidence-based position statement recommending LITT as an established surgical option for brain tumors and RN.^41^

There are several limitations to the present series. While all patients in the cohort had biopsy-proven RN, there is a lack of pathologic data for patients who experienced an additional imaging progression after LITT failure, so the understanding of how LITT may have affected the future evolution of the lesion is limited. Additionally, follow-up pathologic data would have helped to address questions surrounding the small, but real sampling error that can occur from stereotactic biopsy. Another limitation was the lack of a comparator group (eg, steroids or bevacizumab) as a means to determine which modality offers the highest therapeutic index in the treatment of RN. The presently accruing REMASTer study, NCT05124912, is randomizing patients with pathologically proven RN to LITT versus steroids, and will hopefully make significant strides in answering this remaining question.^42^

### Conclusion

This prospective multicenter study allowed for the largest to-date analysis of LITT for biopsy-proven RN. LITT offers patients an early diagnosis and a safe and effective minimally invasive treatment option for those with RN. Importantly, LITT allows for the prompt cessation of steroids, the continuation of chemotherapy and/or immunotherapy, excellent control of symptoms, and avoidance of subsequent interventions such as bevacizumab or neurological death from RN.

## Supplementary Material

vdad031_suppl_Supplementary_Figure_S1

vdad031_suppl_Supplementary_Material
