# Supplementary figures and images for: Efficacy of laser interstitial thermal therapy for biopsy-proven radiation necrosis in radiographically recurrent brain metastases
Source: Neurooncol Adv. 2023 Mar 28;5(1):vdad031. doi: 10.1093/noajnl/vdad031 (PMC10129388; doi:10.1093/noajnl/vdad031)

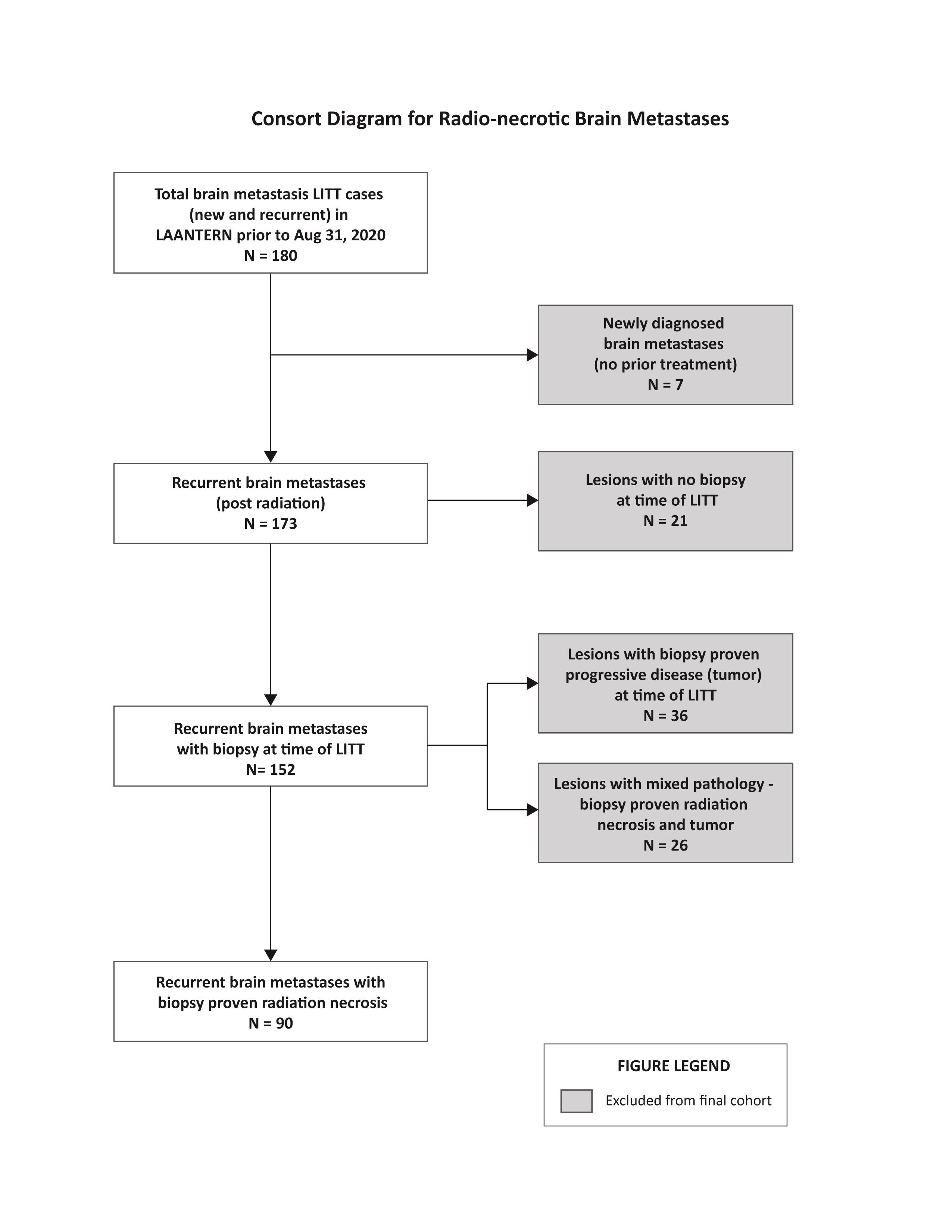

Supplement: vdad031_suppl_Supplementary_Figure_S1 [file vdad031_suppl_supplementary_figure_s1.jpeg]
