# Supplementary material for: Efficacy of laser interstitial thermal therapy for biopsy-proven radiation necrosis in radiographically recurrent brain metastases
Source: Neurooncol Adv. 2023 Mar 28;5(1):vdad031. doi: 10.1093/noajnl/vdad031 (PMC10129388; doi:10.1093/noajnl/vdad031)
Supplement: vdad031_suppl_Supplementary_Material [file vdad031_suppl_supplementary_material.docx]

# Supplementary Material:

**Supplementary Table 1: Karnofsky Performance scale Changes Over Time**

| **Time of assessment**  **N, Mean ± SD, Median** | **Visit** | **Change relative to baseline**  **Mean ± SD, p value** |
| --- | --- | --- |
| Baseline | 81  83.6 ± 14.1  90.0 | - |
| 1 Month | 66  82.3 ± 14.0  80.0 | -3.3 ± 10.9  0.018 |
| 3 Month | 53  81.3 ± 15.2  80.0 | -3.8 ± 13.2  0.048 |
| 6 Month | 50  81.8 ± 14.2  80.0 | -5.8 ± 12.7  0.003 |
| 1 Year | 40  80.3 ± 16.7  80.0 | -6.2 ± 18.0  0.043 |
| 2 Year | 23  82.2 ± 12.0  80.0 | -5.2 ± 13.6  0.094 |

**Supplementary Table 2: FACT Br Total Score Over Time**

| **FACT Br Total Score** |  |  |
| --- | --- | --- |
| Baseline |  |  |
| N | 82 |  |
| Mean ± SD | 143.7 ± 26.4 |  |
| Median | 141.0 |  |
| (Min, Max) | (95.0, 196.0) |  |
| 1 Month |  |  |
| N | 69 | 66 |
| Mean ± SD | 144.7 ± 27.9 | -0.9 ± 22.9 |
| Median | 143.0 | 2.0 |
| (Min, Max) | (78.0, 199.0) | (-80.0, 46.0) |
| p-value |  | 0.743 |
| 3 Months |  |  |
| N | 58 | 55 |
| Mean ± SD | 143.7 ± 29.5 | -4.4 ± 22.8 |
| Median | 140.0 | -4.0 |
| (Min, Max) | (85.0, 200.0) | (-64.0, 39.0) |
| p-value |  | 0.159 |
| 6 Months |  |  |
| N | 54 | 50 |
| Mean ± SD | 144.2 ± 30.3 | -5.5 ± 26.2 |
| Median | 144.5 | -4.5 |
| (Min, Max) | (60.0, 198.0) | (-89.0, 52.0) |
| p-value |  | 0.147 |
| 1 Year |  |  |
| N | 47 | 43 |
| Mean ± SD | 150.3 ± 29.6 | 0.1 ± 21.2 |
| Median | 151.0 | -1.0 |
| (Min, Max) | (85.0, 197.0) | (-48.0, 47.0) |
| p-value |  | 0.970 |
| 2 Years |  |  |
| N | 13 | 12 |
| Mean ± SD | 149.8 ± 26.1 | -9.8 ± 23.1 |
| Median | 142.0 | -3.0 |
| (Min, Max) | (114.0, 192.0) | (-67.0, 18.0) |
| p-value |  | 0.172 |

**Supplementary Table 3: Headache Status Over Time**

| **Visit** | **Yes % (n/N)** | **Change better % (n/N)** | **Change worse % (n/N)** | **Reported no change % (n/N)** |
| --- | --- | --- | --- | --- |
| Baseline | 31.4% (27/86) |  |  |  |
| 1 Month | 27.4% (20/73) | 25.0% (5/20) | 25.0% (5/20) | 40.0% (8/20) |
| 3 Months | 23.4% (15/64) | 40.0% (6/15) | 20.0% (3/15) | 20.0% (3/15) |
| 6 Months | 25.5% (14/55) | 14.3% (2/14) | 28.6% (4/14) | 21.4% (3/14) |
| 12 Months | 24.0% (12/50) | 16.7% (2/12) | 16.7% (2/12) | 58.3% (7/12) |
